# A defucosylated anti-PD-L1 monoclonal antibody 13-mG_2a_-f exerts antitumor effects in mouse xenograft models of oral squamous cell carcinoma

**DOI:** 10.1016/j.bbrep.2020.100801

**Published:** 2020-08-30

**Authors:** Junko Takei, Tomokazu Ohishi, Mika K. Kaneko, Hiroyuki Harada, Manabu Kawada, Yukinari Kato

**Affiliations:** a Department of Antibody Drug Development, Tohoku University Graduate School of Medicine, 2-1 Seiryo-machi, Aoba-ku, Sendai, Miyagi, 980-8575, Japan; b Department of Oral and Maxillofacial Surgery, Graduate School of Medical and Dental Sciences, Tokyo Medical and Dental University, 1-5-45, Yushima, Bunkyo-ku, Tokyo, 113-8510, Japan; c Institute of Microbial Chemistry (BIKAKEN), Numazu, Microbial Chemistry Research Foundation, 18-24 Miyamoto, Numazu-shi, Shizuoka, 410-0301, Japan; d New Industry Creation Hatchery Center, Tohoku University, 2-1, Seiryo-machi, Aoba-ku, Sendai, Miyagi, 980-8575, Japan

**Keywords:** PD-L1, Monoclonal antibody, ADCC, CDC, Antitumor activity, Oral cancer

## Abstract

Programmed cell death ligand-1 (PD-L1) is a type I transmembrane glycoprotein expressed on antigen-presenting cells and several tumor cells, including melanoma and lung cancer cells. A strong correlation has been reported between PD-L1 expression in tumor cells and negative prognosis in cancer patients. Previously, we established an anti-PD-L1 monoclonal antibody (mAb), L_1_Mab-13 (IgG_1_, kappa), by immunizing mice with PD-L1-overexpressing CHO-K1 cells. L_1_Mab-13 specifically reacts with endogenous PD-L1 in lung cancer cell lines in flow cytometry and Western blot applications, and stains a plasma membrane-like pattern in lung cancer tissues via immunohistochemical analysis. In this study, we investigated whether L_1_Mab-13 reacts with oral cancer cell lines and exerts antitumor activities. Because L_1_Mab-13 lacks antibody-dependent cellular cytotoxicity (ADCC) and complement-dependent cytotoxicity (CDC), we first converted the subclass of L_1_Mab-13 from IgG_1_ into IgG_2a_ (13-mG_2a_), and further produced a defucosylated version (13-mG_2a_-f) using FUT8-deficient ExpiCHO-S (BINDS-09) cells. Defucosylation of 13-mG_2a_-f was confirmed using fucose-binding lectins, such as *Aleuria aurantia* and *Pholiota squarrosa* lectins. The dissociation constants (*K*_D_) for 13-mG_2a_-f in SAS and HSC-2 oral cancer cells were determined via flow cytometry to be 2.8 × 10^−9^ M and 4.8 × 10^−9^ M, respectively, indicating that 13-mG_2a_-f possesses extremely high binding affinity. *In vitro* analysis demonstrated that 13-mG_2a_-f showed moderate ADCC and CDC activities against SAS and HSC-2 oral cancer cells. *In vivo* analysis revealed that 13-mG_2a_-f significantly reduced tumor development in SAS and HSC-2 xenografts in comparison to control mouse IgG, even after injection seven days post-tumor inoculation. Taken together, these data demonstrate that treatment with 13-mG_2a_-f may represent a useful therapy for patients with PD-L1-expressing oral cancers.

## Highlights

• ・PD-L1 is expressed in many tumor cells including oral cancers.
• ・We previously established an anti-PD-L1 mAb (L_1_Mab-13, mouse IgG_1_).
• ・We converted the subclass of L_1_Mab-13 from IgG_1_ into IgG_2a_ (13-mG_2a_), and defucosylated it (13-mG_2a_-f).
• ・13-mG_2a_-f exerted ADCC, CDC, and antitumor activities against oral cancer cell lines.

## Abbreviations:

AAL: *Aleuria aurantia* lectin
ADCC: antibody-dependent cellular cytotoxicity
ATCC: American Type Culture Collection
BSA: bovine serum albumin
CasMab: cancer-specific mAb
CDC: complement-dependent cytotoxicity
CHO: Chinese hamster ovary
Con A: concanavalin A
DMEM: Dulbecco's Modified Eagle's Medium
EDTA: ethylenediaminetetraacetic acid
ELISA: enzyme-linked immunosorbent assay
FBS: fetal bovine serum
HNSCC: head and neck squamous cell carcinoma
JCRB: Japanese Collection of Research Bioresources Cell Bank
mAb: monoclonal antibody
OSCC: oral squamous cell carcinoma
PBS: phosphate-buffered saline
PDPN: podoplanin
PhoSL: *Pholiota squarrosa* lectin
PODXL: podocalyxin
SCC: squamous cell carcinoma

## 1 Introduction

Programmed cell death ligand-1 (PD-L1) is a type I transmembrane glycoprotein expressed in many tumor types, including melanoma, brain tumor, lung cancer, breast cancer, gastric cancer, ovarian cancer, pancreatic cancer, and renal cancer [[1], [2], [3], [4], [5], [6], [7]]. PD-L1 is an immune regulatory molecule, which limits T cell effector function [8]. Programmed cell death (PD)-1/PD-L1 association inhibits activated T cell proliferation, allowing cancer cells to circumvent host immune surveillance [9,10]. A substantial correlation between PD-L1/L2 expression in cancer cells and poor prognosis has been reported in several cancers [[11], [12], [13]]. Although inhibition of PD-1 in patients with tumors is demonstrated to have therapeutic effects, detecting PD-L1 expression via immunohistochemistry would be extremely beneficial in making the clinical determination to use targeted drugs such as nivolumab or pembrolizumab for cancer treatment [14,15].

Oral cancers occupy about 2% of all cancer cases worldwide [16]. More than 350,000 individuals are diagnosed as oral cancer every year, and oral cancers are fatal for about 170,000 of these people. Increasingly, young patients are being diagnosed with oral cancers [17,18]. Oral cancers comprise several histological tumor types, such as squamous cell carcinoma (SCC), adenocarcinoma, mucoepidermoid carcinoma, and adenoid cystic carcinoma. The most effective treatment of oral SCC (OSCC), which comprises over 90% of all oral cancers, depends upon its clinical stage [19]. Early stages (stage-I and –II) are treated by surgery or radiotherapy alone. In contrast, advanced stages (stage-III and -IV) require a combination of surgery, radiotherapy, and chemotherapy [20]. For chemotherapy of OSCCs, cisplatin is the primary drug of choice, and it is usually combined with 5-fluorouracil and docetaxel [21,22]. Other anticancer agents, such as paclitaxel, methotrexate, and carboplatin can be also used for OSCCs [23], but effective molecular targeting drugs, including antibody therapies, are lacking.

PD-L1 has been utilized not only as a molecular marker of anti-PD-1 therapy, but also as a molecular target for antibody therapy. Anti-PD-L1 mAbs, such as atezolizumab, durvalumab, and avelumab has been used for patients with advanced head and neck squamous cell carcinoma (HNSCC) [24]. In 45%–87% of OSCC cases, cancer cells were PD-L1 positive, depending on the cut-off value for positivity and whether cytoplasmic staining was included as positive [24]. Anti-PD-L1 mAbs have been mainly used for PD1/PD-L1 blockade, but antitumor activities by antibody-dependent cellular cytotoxicity (ADCC) and complement-dependent cytotoxicity (CDC) against oral cancers have not been investigated.

In our previous study, we developed a novel anti-human PD-L1 antibody, L_1_Mab-13 (mouse IgG_1_, kappa), which is useful for flow cytometry, Western blot, and immunohistochemical analysis [25]. In this study, we converted IgG_1_ subclass L_1_Mab-13 into IgG_2a_ subclass 13-mG_2a_, and further produced a defucosylated version, 13-mG_2a_-f, using FUT8-deficient ExpiCHO-S cells (BINDS-09) [26]. We then investigated whether 13-mG_2a_-f exhibited ADCC, CDC, and antitumor activities against oral cancers.

## 2 Materials and methods

### 2.1 Cell lines

CHO-K1 was obtained from the American Type Culture Collection (ATCC, Manassas, VA). CHO/PD-L1 was previously established [25]. Oral squamous carcinoma cell lines, including SAS (tongue) and HSC-2 (oral cavity), were obtained from the Japanese Collection of Research Bioresources Cell Bank (JCRB, Osaka, Japan). CHO-K1 and CHO/PD-L1 were cultured in RPMI 1640 medium (Nacalai Tesque, Inc., Kyoto, Japan). SAS and HSC-2 were cultured in Dulbecco's modified Eagle's medium (DMEM; Nacalai Tesque, Inc.). The medium is supplemented with 10% heat-inactivated fetal bovine serum (FBS; Thermo Fisher Scientific Inc., Waltham, MA, USA), 100 units/mL of penicillin, 100 μg/mL streptomycin, and 0.25 μg/mL amphotericin B (Nacalai Tesque, Inc.) at 37 °C in a humidified atmosphere containing 5% CO_2_.

### 2.2 Antibodies

Anti-PD-L1 mAb L_1_Mab-13 (mouse IgG_1_, kappa) was developed as previously described [25]. To generate 13-mG_2a_, appropriate V_H_ cDNA of mouse L_1_Mab-13 and C_H_ of mouse IgG_2a_ were subcloned into pCAG-Ble vector (FUJIFILM Wako Pure Chemical Corporation, Osaka, Japan), and V_L_ and C_L_ cDNAs of L_1_Mab-13 were subcloned into pCAG-Neo vector (FUJIFILM Wako Pure Chemical Corporation). The vectors were transfected into BINDS-09 (FUT8-deficient ExpiCHO-S cells) using the ExpiCHO Expression System [26]. 13-mG_2a_-f was purified using Protein G-Sepharose (GE Healthcare Bio-Sciences, Pittsburgh, PA). Mouse IgG was purchased from Sigma-Aldrich Corp. (St. Louis, MO).

### 2.3 Flow cytometry

Cells were harvested by brief exposure to 0.25% trypsin/1 mM ethylenediaminetetraacetic acid (EDTA; Nacalai Tesque, Inc.). After washing with 0.1% bovine serum albumin (BSA) in phosphate-buffered saline (PBS), cells were treated with primary mAbs for 30 min at 4 °C and subsequently with Alexa Fluor 488-conjugated anti-mouse IgG (1:1000; Cell Signaling Technology, Inc., Danvers, MA, USA). Fluorescence data were collected using an EC800 Cell Analyzer (Sony Corp., Tokyo, Japan).

### 2.4 Determination of the binding affinity

Cells were suspended in 100 μL of serially diluted mAbs (0.3 ng/mL–5 μg/mL), followed by the addition of Alexa Fluor 488-conjugated anti-mouse IgG (1:200; Cell Signaling Technology, Inc.). Fluorescence data were collected using an EC800 Cell Analyzer (Sony Corp.). The dissociation constant (*K*_D_) was calculated by fitting binding isotherms to built-in one-site binding models in GraphPad PRISM 6 (GraphPad Software, Inc., La Jolla, CA, USA).

### 2.5 Enzyme-linked immunosorbent assay (ELISA)

L_1_Mab-13 and 13-mG_2a_-f were immobilized on Nunc Maxisorp 96-well immunoplates (Thermo Fisher Scientific Inc.) at 1 μg/mL for 30 min. After blocking using SuperBlock buffer (Thermo Fisher Scientific Inc.) containing 0.5 mM CaCl_2_, the plates were incubated with biotin-labeled lectins, such as *Aleuria aurantia* lectin (AAL, fucose binder; Vector Laboratories, Burlingame, CA, USA) [27], *Pholiota squarrosa* lectin (PhoSL, core fucose binder; J-OIL MILLS, Inc., Tokyo, Japan) [28], and Concanavalin A (ConA, mannose binder; Vector Laboratories) [29], followed by 1:3000 diluted peroxidase-conjugated streptavidin (Agilent Technologies, Santa Clara, CA, USA). The enzymatic reaction was produced using a 1-Step Ultra TMB-ELISA (Thermo Fisher Scientific Inc.). Optical density was measured at 655 nm using an iMark microplate reader (Bio-Rad Laboratories, Inc., Berkeley, CA, USA).

### 2.6 Animals

All animal experiments were performed in accordance with relevant guidelines and regulations to minimize animal suffering and distress in the laboratory. Animal studies for ADCC and antitumor activity were approved by the institutional committee for experiments of the Institute of Microbial Chemistry (Permit number: 2020–007). Mice were monitored for health and weight every 2 or 4 days. Experiment duration was 3 weeks. A bodyweight loss exceeding 25% and a maximum tumor size exceeding 3000 mm^3^ were identified as humane endpoints. Mice were euthanized by cervical dislocation, and the death was verified by respiratory arrest and cardiac arrest.

### 2.7 ADCC

Six 6-week-old female BALB/c nude mice were purchased from Charles River (Kanagawa, Japan). After euthanization by cervical dislocation, spleens were removed aseptically and single-cell suspensions obtained by forcing spleen tissues through a sterile cell strainer (352360, BD Falcon, Corning, New York, NY, USA) using a syringe. Erythrocytes were lysed with a 10-sec exposure to ice-cold distilled water. Splenocytes were washed with DMEM and resuspended in DMEM with 10% FBS and used as effector cells. Target cells were labeled with 10-μg/mL Calcein AM (Thermo Fisher Scientific, Inc.) and resuspended in the same medium. The target cells (2 × 10^4^ cells/well) were plated in 96-well plates and mixed with effector cells, anti-PD-L1 antibodies, or control IgG (mouse IgG_2a_) (Sigma-Aldrich). After a 6.5-h incubation, the Calcein AM release of supernatant from each well was measured. Fluorescence intensity was determined using a microplate reader (Power Scan HT) (BioTek Instruments, Winooski, VT, USA) with an excitation wavelength of 485 nm and an emission wavelength of 538 nm. Cytolytic activity (as % of lysis) was calculated using the equation: % lysis = (E−S)/(M−S) × 100, where E is fluorescence of combined target and effector cells, S is spontaneous fluorescence of target cells only, and M is maximum fluorescence measured after lysing all cells with a buffer containing 0.5% Triton X-100, 10 mM Tris-HCl (pH 7.4), and 10 mM of EDTA.

### 2.8 CDC

To assess cell viability, cells were labeled with 10-μg/mL Calcein AM (Thermo Fisher Scientific, Inc.) and resuspended in the same medium. The cells (2 × 10^4^ cells/well) were plated in 96-well plates and mixed with rabbit complement (final dilution 1:10; Low-Tox-M Rabbit Complement; Cedarlane Laboratories, Hornby, Ontario, Canada), anti-PD-L1 antibodies, or control IgG (mouse IgG_2a_) (Sigma-Aldrich Corp.). After a 6.5-h incubation, the Calcein AM release of supernatant from each well was measured. Fluorescence intensity was determined as described in the ADCC part above.

### 2.9 Antitumor activity of 13-mG_2a_-f in the xenografts of oral cancers

Thirty-two 6-week-old female BALB/c nude mice were purchased from Charles River (Kanagawa, Japan) and used at 7 weeks of age. SAS and HSC-2 cells (0.3 mL of 1.33 × 10^8^ cells/mL in DMEM) were mixed with 0.5 mL BD Matrigel Matrix Growth Factor Reduced (BD Biosciences, San Jose, CA, USA). 100 μL of this suspension (5 × 10^6^ cells) was injected subcutaneously into the left flank. After day 8, 100 μg of 13-mG_2a_-f and control mouse IgG (Sigma-Aldrich Corp.) in 100 μL PBS was injected intraperitoneally (i.p.) into treated and control mice, respectively. Additional antibodies were then injected on days 14 and 21. Twenty-three days after cell implantation, all mice were euthanized by cervical dislocation, and tumor diameters and volumes were determined as previously described [30].

### 2.10 Statistical analyses

All data were expressed as mean ± SEM. Statistical analysis used ANOVA and subsequently Sidak's multiple comparisons test for tumor volume and mouse weight, or Welch's *t*-test for ADCC/CDC and tumor weight using GraphPad Prism 7 (GraphPad Software, Inc.). *P* < 0.05 was adopted as a level of statistical significance.

## 3 Results

### 3.1 Development and characterization of 13-mG_2a_-f, a core-fucose-deficient mouse IgG_2a_-type anti-PD-L1 antibody

**Fig. 1:**
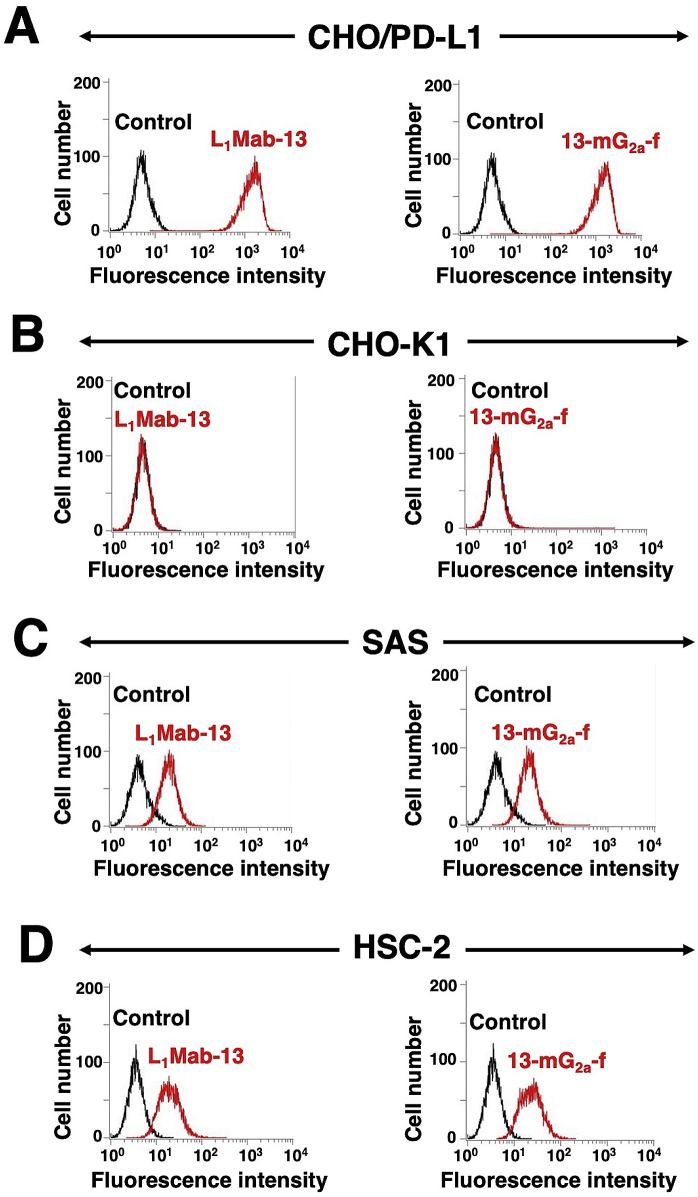
Recognition of PD-L1 using anti-PD-L1 mAbs. (A) CHO/PD-L1 cells were treated with L_1_Mab-13 and 13-mG_2a_-f (1 μg/mL), followed by secondary antibodies. (B) CHO-K1 cells were treated with L_1_Mab-13 and 13-mG_2a_-f (1 μg/mL), followed by secondary antibodies. (C) SAS cells were treated with L_1_Mab-13 and 13-mG_2a_-f (1 μg/mL), followed by secondary antibodies. (D) HSC-2 cells were treated with L_1_Mab-13 and 13-mG_2a_-f (1 μg/mL), followed by secondary antibodies. The black line represents the negative control.

As mouse IgG_2a_ possesses high ADCC and CDC activities [31], we first developed a mouse IgG_2a_ version of L_1_Mab-13 (mouse IgG_1_) by subcloning appropriate V_H_ cDNA of L_1_Mab-13 and C_H_ of mouse IgG_2a_ into pCAG-Ble vector, and the light chain of L_1_Mab-13 into pCAG-Neo vector. This IgG_2a_-type of L_1_Mab-13 is henceforth referred to as 13-mG_2a_. We additionally produced a core-fucose-deficient type of 13-mG_2a_, henceforth referred to as 13-mG_2a_-f, using the BINDS-09 cell line (FUT8-knockout ExpiCHO–S cell line) [26]. We analyzed the sensitivity of 13-mG_2a_-f in CHO-K1 cells expressing PD-L1 (CHO/PD-L1) and in OSCC cell lines (SAS and HSC-2) using flow cytometry. Both L_1_Mab-13 and 13-mG_2a_-f reacted with CHO/PD-L1 cells (Fig. 1A), but not with CHO-K1 cells (Fig. 1B). Both mAbs also reacted with SAS cells (Fig. 1C) and HSC-2 cells (Fig. 1D), indicating that 13-mG_2a_-f demonstrated high sensitivity and specificity for PD-L1.

**Fig. 2:**
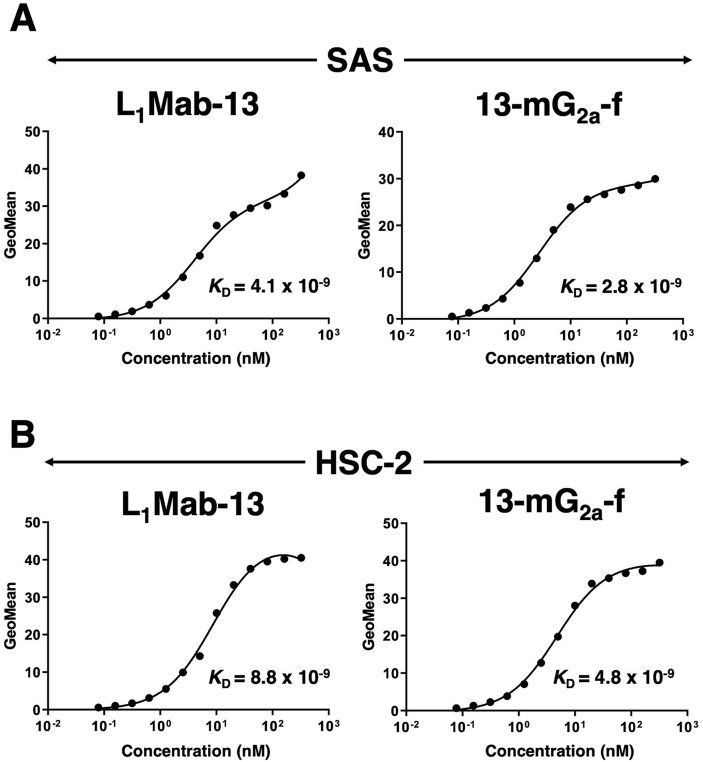
Determination of the binding affinity of anti-PD-L1 mAbs for oral cancer cells using flow cytometry. (A) SAS cells were suspended in 100 μl of serially diluted mAbs (0.3 ng/mL - 5 μg/mL), followed by the addition of Alexa Fluor 488-conjugated anti-mouse IgG, and fluorescence data were collected. (B) HSC-2 cells were suspended in 100 μL of serially diluted mAbs (0.3 ng/mL - 5 μg/mL), followed by the addition of Alexa Fluor 488-conjugated anti-mouse IgG, and fluorescence data were collected.

We conducted a kinetic analysis of the interactions of L_1_Mab-13 and 13-mG_2a_-f with SAS and HSC-2 oral cancer cell lines using flow cytometry. The dissociation constant (*K*_D_) for L_1_Mab-13 in SAS cells was determined to be 4.1 × 10^−9^ M (Fig. 2A). In contrast, the *K*_D_ for 13-mG_2a_-f in SAS cells was 2.8 × 10^−9^ M. The binding affinity of 13-mG_2a_-f in SAS cells was 1.5-fold higher than that of L_1_Mab-13. Likewise, the *K*_D_ for L_1_Mab-13 against HSC-2 cells was 8.8 × 10^−9^ M (Fig. 2B). By contrast, the *K*_D_ for 13-mG_2a_-f in HSC-2 cells was 4.8 × 10^−9^ M. The binding affinity of 13-mG_2a_-f in HSC-2 cells was 1.8-fold higher than that of L_1_Mab-13. The binding affinity of 13-mG_2a_-f in SAS cells was 1.7-fold higher than that in HSC-2 cells.

**Fig. 3:**
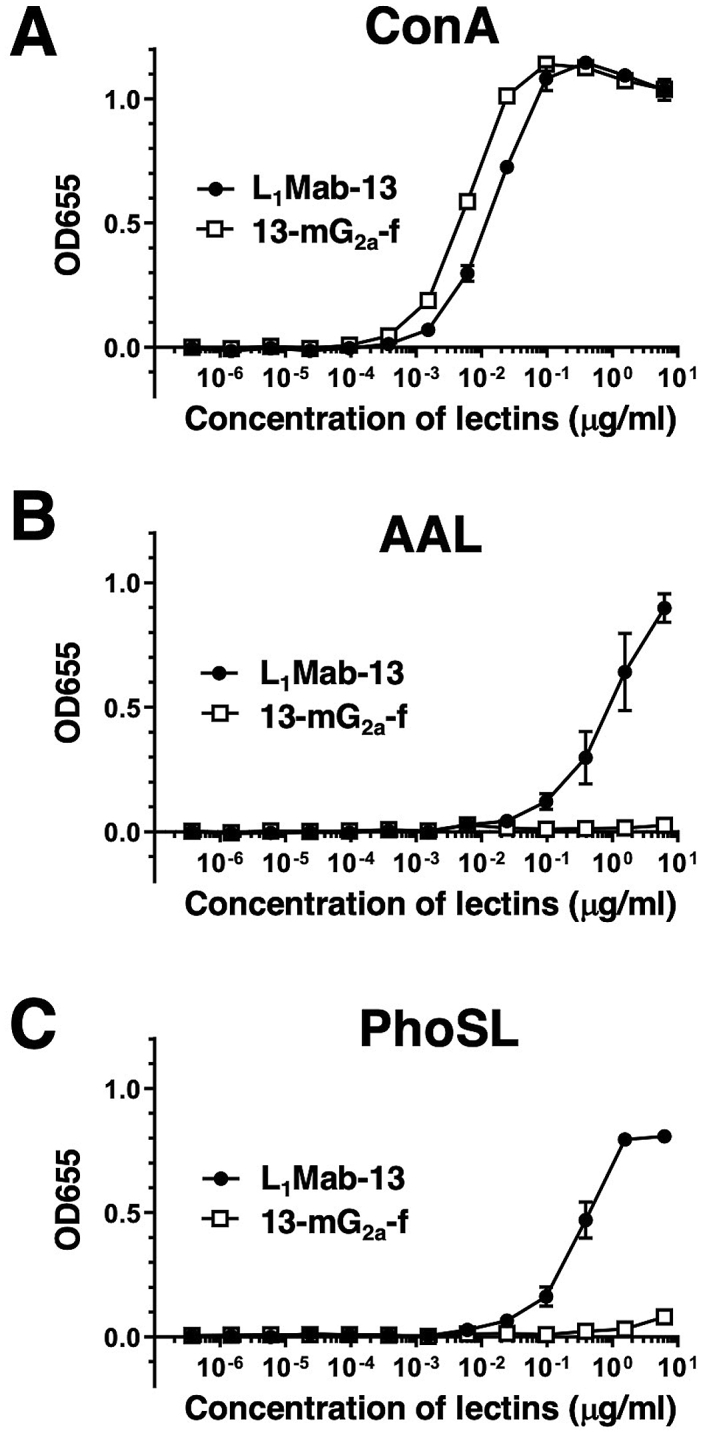
Confirmation of defucosylation of 13-mG_2a_-f by enzyme-linked immunosorbent assay (ELISA) using lectins. (A) L_1_Mab-13 and 13-mG_2a_-f were immobilized and incubated with biotin-labeled concanavalin A (Con A), followed by peroxidase-conjugated streptavidin. OD_655_ was measured as a function of Con A concentration. (B) L_1_Mab-13 and 13-mG_2a_-f were immobilized and incubated with biotin-labeled *Aleuria aurantia* lectin (AAL), followed by peroxidase-conjugated streptavidin. OD_655_ was measured as a function of AAL concentration. (C) L_1_Mab-13 and 13-mG_2a_-f were immobilized and incubated with biotin-labeled *Pholiota squarrosa* lectin (PhoSL), followed by peroxidase-conjugated streptavidin. OD_655_ was measured as a function of PhoSL concentration.

Defucosylation of 13-mG_2a_-f was confirmed using lectins such as AAL (which binds to fucose) and PhoSL (which binds to core fucose). ConA (which binds to mannose) was used as a positive control. Both L_1_Mab-13 and 13-mG_2a_-f were detected using ConA (Fig. 3A). L_1_Mab-13, but not 13-mG_2a_-f, was detected using AAL (Fig. 3B) and PhoSL (Fig. 3C), demonstrating that 13-mG_2a_-f was defucosylated.

### 3.2 ADCC and CDC activities of 13-mG_2a_-f in oral cancer cell lines

**Fig. 4:**
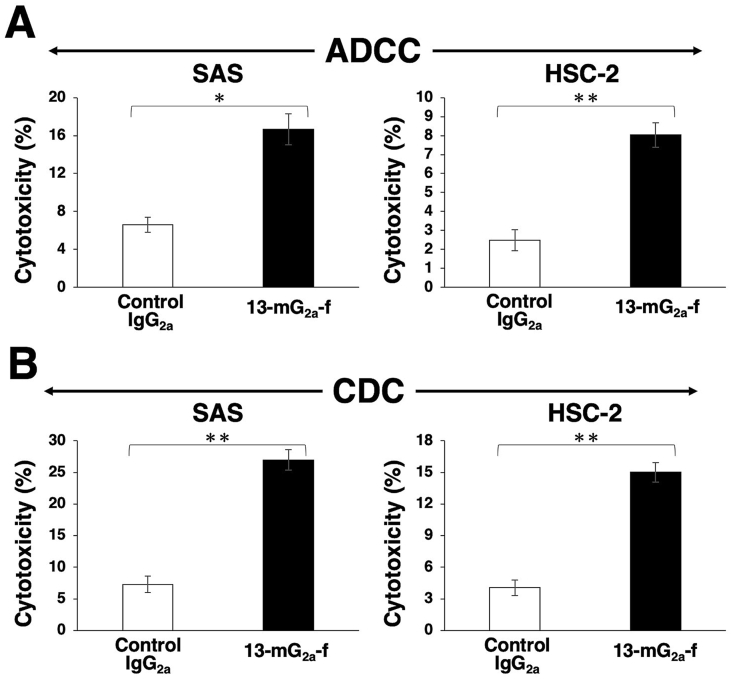
Evaluation of ADCC and CDC activities of 13-mG_2a_-f in SAS and HSC-2 cells. (A) ADCC activities of 13-mG_2a_-f and control mouse IgG_2a_ in SAS and HSC-2 cells. (B) CDC activities by 13-mG_2a_-f and control mouse IgG_2a_ in SAS and HSC-2 cells. Values are mean ± SEM. Asterisk indicates statistical significance (**P < 0.01, *P < 0.05, n.s: not significant, Welch's *t*-test).

Because the mouse IgG_1_ subclass L_1_Mab-13 does not possess ADCC or CDC activities, we created a mouse IgG_2a_ subclass mAb, and further defucosylated it to enhance those activities. In this study, we examined whether the developed 13-mG_2a_-f induced ADCC and CDC in PD-L1-expressing oral cancer cell lines, such as SAS and HSC-2 cells. 13-mG_2a_-f exhibited higher ADCC (17% cytotoxicity) in SAS cells compared with that of control mouse IgG_2a_ treatment (6.6% cytotoxicity; *P* < 0.05) (Fig. 4A). Similarly, 13-mG_2a_-f exhibited higher ADCC (8.0% cytotoxicity) in HSC-2 cells compared with that of control mouse IgG_2a_ treatment (2.5% cytotoxicity; *P* < 0.01) (Fig. 4A).

Furthermore, 13-mG_2a_-f exhibited higher CDC activity (27% cytotoxicity) in SAS cells compared with control mouse IgG_2a_ treatment (7.3% cytotoxicity; *P* < 0.01; Fig. 4B). Similarly, 13-mG_2a_-f exhibited higher CDC activity (15% cytotoxicity) in HSC-2 cells compared with control mouse IgG_2a_ treatment (4.1% cytotoxicity; *P* < 0.01; Fig. 4B). Although ADCC/CDC activities of 13-mG_2a_-f in oral cancer cells are not outstanding, it remained to be seen whether 13-mG_2a_-f may exert antitumor activity against oral cancer cells *in vivo*.

### 3.3 Antitumor activities of 13-mG_2a_-f in the mouse xenografts of SAS oral cancer cells

**Fig. 5:**
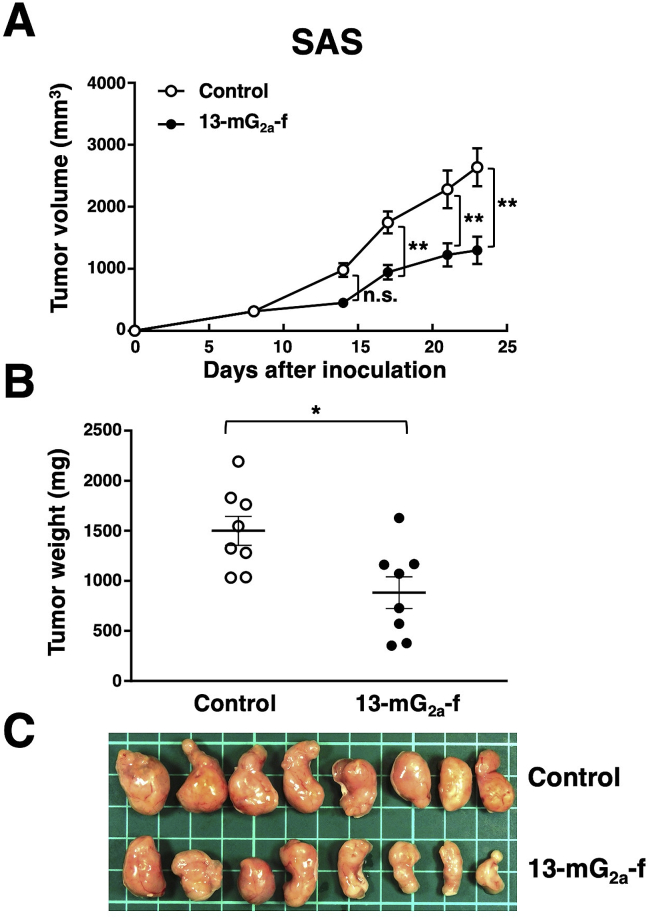
Evaluation of antitumor activity of 13-mG_2a_-f in SAS xenografts. (A) Tumor volume of SAS xenografts. SAS cells (5 × 10^6^ cells) were injected subcutaneously into the left flank. After eight days, 100 μg of 13-mG_2a_-f or control mouse IgG in 100 μl PBS were injected i.p. into treated and control mice, respectively. Additional antibodies were then injected on days 14 and 21. Tumor volume was measured on days 8, 14, 17, 21, and 23. Values are mean ± SEM. Asterisk indicates statistical significance (**P < 0.01, n.s: not significant, ANOVA and Sidak's multiple comparisons test) (B) Tumor weights of SAS xenografts. Tumors of SAS xenografts were resected from 13-mG_2a_-f and control mouse IgG groups. Tumor weight on day 23 was measured from excised xenografts. Values are mean ± SEM. Asterisk indicates statistical significance (*P < 0.05, Welch's *t*-test). (C) Resected tumors of SAS xenografts from 13-mG_2a_-f and control mouse IgG groups on day 23.

In the SAS xenograft models, tumor formation in 16 SAS-injected mice was observed after eight days. These 16 SAS tumor-bearing mice were then divided into a 13-mG_2a_-f-treated group and a control group. On days 8, 14, and 21 after SAS cell injections into the mice, 13-mG_2a_-f (100 μg) and control mouse IgG (100 μg) were injected i.p. into the mice. Tumor formation was observed in mice in both treated and control groups. Tumor volume was measured on days 8, 14, 17, 21, and 23 after SAS cell injection. 13-mG_2a_-f-treated mice showed significantly reduced tumor development on day 17 (*P* < 0.01), day 21 (*P* < 0.01), and day 23 (*P* < 0.01) in comparison to IgG-treated control mice (Fig. 5A). Tumor volume reduction by 13-mG_2a_-f treatment was 51% on day 23. Tumors from 13-mG_2a_-f-treated mice weighed significantly less than tumors from IgG-treated control mice (41% reduction, *P* < 0.05; Fig. 5B). Resected tumors on day 23 are depicted (Fig. 5C). Total body weights did not significantly differ between the two groups (data not shown). These results indicate that 13-mG_2a_-f reduced the growth of SAS xenografts effectively, even when 13-mG_2a_-f was injected eight days post-SAS cell injections in mice.

### 3.4 Antitumor activities of 13-mG_2a_-f in mouse xenografts of HSC-2 oral cancer cells

**Fig. 6:**
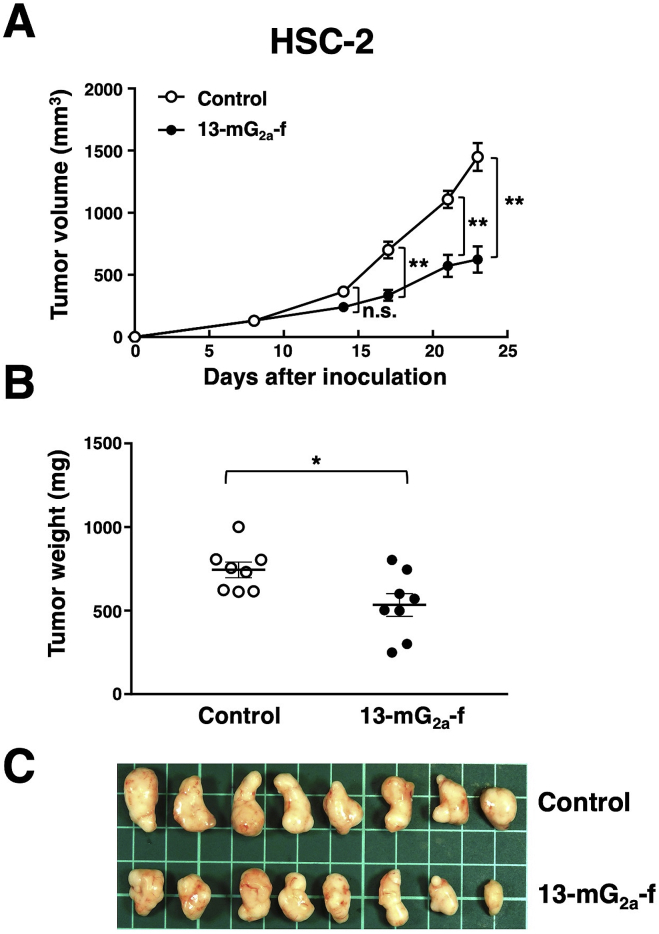
Evaluation of antitumor activity of 13-mG_2a_-f in HSC-2 xenografts. (A) Tumor volume of HSC-2 xenografts. HSC-2 cells (5 × 10^6^ cells) were injected subcutaneously into the left flank. After eight days, 100 μg of 13-mG_2a_-f or control mouse IgG in 100 μl PBS were injected i.p. into treated and control mice, respectively. Additional antibodies were then injected on days 14 and 21. Tumor volume was measured on days 8, 14, 17, 21, and 23. Values are mean ± SEM. Asterisk indicates statistical significance (**P < 0.01, n.s: not significant, ANOVA and Sidak's multiple comparisons test) (B) Tumor weights of HSC-2 xenografts. Tumors of HSC-2 xenografts were resected from 13-mG_2a_-f and control mouse IgG groups. Tumor weight on day 23 was measured from excised xenografts. Values are mean ± SEM. Asterisk indicates statistical significance (*P < 0.05, Welch's *t*-test). (C) Resected tumors of HSC-2 xenografts from 13-mG_2a_-f and control mouse IgG groups on day 23.

In the HSC-2 xenograft models, tumor formation of 16 HSC-2-injected mice was observed after eight days. These 16 HSC-2-bearing mice were then divided into a 13-mG_2a_-f-treated group and a control group. On days 8, 14, and 21 after cell injections into the mice, 13-mG_2a_-f (100 μg) and control mouse IgG (100 μg) were injected i.p. into the mice. Tumor volume was assessed on days 8, 14, 17, 21, and 23. 13-mG_2a_-f-treated mice showed significantly reduced tumor development on day 17 (*P* < 0.01), day 21 (*P* < 0.01), and day 23 (*P* < 0.01) in comparison to IgG-treated control mice (Fig. 6A). Tumor volume reduction in 13-mG_2a_-treated mice was 57% on day 23. Tumors from 13-mG_2a_-f-treated mice weighed significantly less than tumors from IgG-treated control mice (28% reduction, *P* < 0.05; Fig. 6B). Resected tumors on day 23 are shown in Fig. 6C. Total body weights did not significantly differ between the two groups (data not shown). These results indicate that 13-mG_2a_-f reduced the growth of HSC-2 xenografts effectively, even when 13-mG_2a_-f was injected eight days post–HSC–2 cell injections in mice.

## 4 Discussion

In this study, we investigated whether anti-PD-L1 mAbs are advantageous for the treatment of oral cancers by ADCC/CDC activities, rather than by neutralization of PD-L1/PD1 interaction, as human PD-L1 does not react with mouse PD-1. We had previously developed a sensitive and specific anti-PD-L1 mAb, L_1_Mab-13 [25], but were unable to investigate antitumor activity as the IgG_1_ subclass does not possess ADCC/CDC activities. Therefore, we converted L_1_Mab-13 into an IgG_2a_ subclass antibody, and increased ADCC activity via defucosylation. We demonstrated that 13-mG_2a_-f exerts ADCC/CDC activities *in vitro* (Fig. 4), and antitumor activities against oral cancer xenografts *in vivo* (Fig. 5, Fig. 6). Importantly, 13-mG_2a_-f efficaciously reduced the growth of SAS xenografts (Fig. 5) and HSC-2 xenografts (Fig. 6), even when 13-mG_2a_-f was injected eight days after cell implantations into the mice. However, SAS and HSC-2 tumor volume reduction on day 23 by 13-mG_2a_-f treatment only reached 51% and 57%, respectively, indicating that anti-PD-L1 therapy might not be sufficient for solo treatment of most oral cancers. One potential reason for this weak antitumor activity is low ADCC activity (Fig. 4A) and CDC activity (Fig. 4B) of 13-mG_2a_-f, in spite of the high binding affinity in SAS cells (*K*_D_: 2.8 × 10^−9^ M; Fig. 2A) and HSC-2 cells (*K*_D_: 4.8 × 10^−9^ M; Fig. 2B). The binding affinity of 13-mG_2a_-f in SAS cells was 1.5-fold higher than that of L_1_Mab-13. Likewise, the binding affinity of 13-mG_2a_-f in HSC-2 cells was 1.8-fold higher than that of L_1_Mab-13. These results are consistent with our previous observation that the mouse IgG_2a_-type mAb 47-mG_2a_-f also shows a higher affinity for PODXL than the original PcMab-47 (mouse IgG_1_), indicating that fragment crystallizable (Fc) portion of mouse IgG could be important for the binding affinity for target molecules; or, recombinant mAbs might possess high purity compared to hybridoma-derived mAbs [32].

We recently developed a sensitive and specific mAb against EGFR (clone EMab-17, mouse IgG_2a_), and examined its ADCC/CDC and antitumor activities against SAS and HSC-2 xenografts [30]. In another recent study, HER2 was shown to be expressed in oral cancers, and an anti-HER2 mAb (clone H_2_Mab-19, mouse IgG_2b_) showed antitumor activity against SAS and HSC-2 xenografts [33]. Further, we previously investigated whether PODXL may be a therapeutic target in OSCC using anti-PODXL mAbs [32]. We converted an anti-PODXL mAb of IgG_1_ subclass (PcMab-47) into a mouse IgG_2a_-type mAb (47-mG_2a_) to increase ADCC. We developed 47-mG_2a_-f, a core fucose-deficient variant of 47-mG_2a_ to increase its ADCC. *In vivo* analysis demonstrated that 47-mG_2a_-f, but not 47-mG_2a_, exerted antitumor activity in SAS and HSC-2 xenograft models at a dose of 100 μg/mouse/week administered three times. Although both 47-mG_2a_ and 47-mG_2a_-f exhibited antitumor activity in HSC-2 xenograft models at a dose of 500 μg/mouse/week administered twice, 47-mG_2a_-f also demonstrated higher antitumor activity than 47-mG_2a_, indicating that a core fucose-deficient anti-PODXL mAb could be profitable for antibody-based therapy against PODXL-expressing OSCCs.

Targeting multiple targets, such as PODXL, EGFR, HER2, and PD-L1 may be needed for effective therapy to cure oral cancers. Another important goal is to target cancer-specific antigens using a cancer-specific mAb (CasMab). We previously established CasMab against PDPN, which is expressed in many cancers, including oral cancers [34]. In xenograft models with HSC-2 cells, a mouse-human chimeric mAb, chLpMab-23, exerted antitumor activity using human natural killer cells, indicating that chLpMab-23 may be advantageous for antibody therapy against PDPN-expressing oral cancers [35]. In the future, cancer-specific anti-PD-L1 mAbs may also be developed that can reduce the adverse effects of traditional antibody therapy.

## Funding

This research was supported in part by Japan 10.13039/100009619Agency for Medical Research and Development (AMED) under Grant Numbers: JP20am0401013 (Y.K.), JP20am0101078 (Y.K.), and JP20ae0101028 (Y.K.).
